# Physical Activity and Health-Related Fitness of Adolescents within the Juvenile Justice System

**DOI:** 10.1155/2018/9710714

**Published:** 2018-07-15

**Authors:** Timothy A. Brusseau, Ryan D. Burns, James C. Hannon

**Affiliations:** ^1^Department of Health, Kinesiology, and Recreation, University of Utah, Salt Lake City, UT, USA; ^2^College of Education, Health, and Human Services, Kent State University, Kent, OH, USA

## Abstract

**Background:**

The purpose of this study was to examine the physical activity patterns and health-related fitness levels of adolescents within the Juvenile Justice System.

**Methods:**

Participants included 68 adolescents (Mean age = 17.1 ± 1.0 years) in two secure Juvenile Justice correctional facilities in the Western USA. Moderate-to-vigorous physical activity (MVPA) was monitored for one week using the ActiGraph GT9X accelerometer. Health-related fitness was measured using the FITNESSGRAM test battery.

**Results:**

Adolescents averaged 43.3±21.6 minutes of MVPA per weekday compared to 42.7±27.5 per weekend day. During school hours, adolescents accumulated 17.1±9.0 minutes of MVPA compared to 5.9±3.4 minutes before school and 21.0±13.6 minutes after school. Adolescents averaged 18.9±11.0 push-ups, 44.5±26.4 curl-ups, 34.7±24.8 PACER laps, and 22.1%±10.0% body fat.

**Conclusions:**

Adolescents within the Juvenile Justice System are falling short of the recommended 60 minutes of MVPA per day and 30 minutes of MVPA during school and also need to improve their health-related fitness, especially cardiorespiratory endurance.

## 1. Introduction

The benefits of physical activity are vital to the physical, psychosocial, and cognitive health of adolescents [1, 2]. A population that may benefit from increases in physical activity and health-related fitness is adolescents within the Juvenile Justice System. In the USA, Juvenile Courts handle an estimated 1.7 million cases each year with more than 110,000 incarcerated in Juvenile Facilities [3, 4]. It is anticipated that more than 70,000 juvenile offenders are in residential placements for adolescents [5], as there are nearly 600 detention facilities in the USA [6, 7]. Juvenile Justice facilities house adolescents with criminal or rehabilitation needs and adolescents can be committed to a facility for a matter of days to more than a year [8]. Given the relatively high prevalence of adolescents within Juvenile Justice facilities in the USA and high length of stay variability, the physical condition of these youth is a public health concern [9, 10]. Implementing interventions within Juvenile Justice System may be an effective way to attenuate health risk via physical activity behavior modification. Indeed, the benefits of daily physical activity participation are numerous as past studies have found relationships between physical activity and physical, psychological, social, and cognitive health indicators in adolescents [11–16].

In addition to the behavior of physical activity, specific components of health-related fitness are important to improve health and wellbeing in youth. Health-related fitness consists of five domains including body composition, cardiorespiratory endurance (aerobic fitness), muscular strength and endurance, and flexibility; however, body composition and cardiorespiratory endurance are the two domains that tend to have the strongest relationships with health outcomes in the pediatric population [17]. Because of the established relationships between body composition, cardiorespiratory endurance, and various health markers, improving these components health-related fitness has become a priority to improve wellbeing and attenuate risk of developing chronic disease [18, 19].

The United States Department of Health and Human Services [20] and the World Health Organization [21] both recommend 60 minutes of moderate-to-vigorous physical activity (MVPA) daily for adolescents (10-19 years old). Achieving 60 minutes of MVPA has shown to be related to the amount of physical activity associated with positive health indicators [11]. Despite the known benefits of MVPA, previous research indicates that adolescents are falling short of both of these recommendations [22–24], which subsequently affects body composition and cardiorespiratory endurance. Furthermore, physical activity and specific components of health-related fitness tend to decline with age [25, 26].

In adult prisoners, there tends to be higher prevalence of unfavorable body composition and cardiorespiratory endurance levels, correlating with length of stay [27, 28]. This suggests that prisoners who have longer sentences tend to have poorer fitness levels compared to prisoners with shorter sentences [27, 28]. Fortunately, physical activity programming has been found to improve psychological wellbeing in male prisoners [29] in addition to improving cardiorespiratory endurance and muscular strength and endurance [30]. In youth, much research in pediatric behavior change, specifically pediatric physical activity programming, has focused on the school environment. However, to our knowledge, little is known regarding the physical activity behavior and the health-related fitness components of body composition, cardiorespiratory endurance, and muscular strength and endurance in incarcerated adolescents within the US Juvenile Justice System. Knowing this information can help devise effective programming. Therefore, the purpose of the study was to explore the physical activity and specific components health-related fitness of adolescents from two secure Juvenile Justice facilities from the state of Utah in the USA.

## 2. Method

### 2.1. Participants

Participants included 68 adolescents (Mean age = 17.1 ± 1.0 years; 60 males, 8 females) incarcerated in two secure Juvenile Justice correctional facilities in the Southwest US Adolescents who were 48% ethnic minority (33% Hispanic, 7% African American, 4% Pacific Islander, 4% American Indian) from one metropolitan area. Adolescents were incarcerated in secure facilities for sentences ranging from 9 months to 2 or more years. A typical weekday had adolescents spending their mornings in their individual or small group units followed by a traditional school schedule before returning to their individual housing units. Gymnasium and field space were available for physical activity although physical activity was not required. Some adolescents if they needed the school credit did have access to physical education (<30%). All procedures were approved by both the University Institutional Review Board and the State Health Research Board. Parents provided written consent and youth provided written assent. Participation was voluntary and the youth could decide to stop participating at any time.

### 2.2. Instruments

#### 2.2.1. Physical Activity

Physical activity was monitored for one week using the ActiGraph (Pensacola, FL) GT9X Link accelerometer. Accelerometer data were recorded in 5-second epochs at 100 Hertz and then processed using Evenson et al. [31] MVPA cut points which have a strong criterion-referenced energy expenditure agreement with indirect calorimetry [32].

#### 2.2.2. Health-Related Fitness

Health-related fitness was assessed using the FITNESSGRAM test battery including push-ups and curl-ups for muscular strength and endurance, the Progressive Aerobic Cardiovascular Endurance Run (PACER) for cardiorespiratory endurance, and two-site skin fold for body composition.

The PACER was administered during an agreed upon time outside of school classes. The PACER was conducted per recommendations [33] across a 20-m distance within an allotted time frame. The final score was recorded in laps.

FITNESSGRAM's push-up test was administered using an audio compact disk providing a cadence of 20 push-ups per minute. The PACER was run by small groups (e.g. 6-12) of students at one time. The 90° push-up is a reliable measure of upper body strength and endurance in children [28]. Push-up scores were recorded as the total number of correctly performed repetitions.

FITNESSGRAM's Dynamic Curl-Up is a test for abdominal muscular endurance. The Dynamic Curl-Up consisted of having the adolescents curl up and down sliding their fingers across a distance of 4.5 inches at a specific cadence provided by a recorded compact disk. Curl-up scores were recorded as total number of correctly performed repetitions [34]. Push-up and curl-up testing were completed in small groups.

Body composition was determined using two-site skinfold assessment with a Lange Skinfold Caliper (Seko, USA). All youth were measured at the right tricep and right medial calf using recommended procedures [34]. The trained Principal Investigator administered the skinfold assessment. Skinfolds were taken in duplicate and averaged across two trials. If two measurements were off by more than 2mm, a third measurement was taken at the respective site. The Slaughter et al. [34] formula was used to estimate body fat percentage. Body composition testing was done individually in a semiprivate area.

### 2.3. Procedures

Accelerometers were distributed each morning by facility staff and youth wore them on their right hip above the iliac crest all day (approximately 6am-10pm). Facility attire included sweat pants which some students suggested made using the accelerometer clip challenging due flimsy nature of the waist band which led to the monitors occasionally falling off; however, wear time did not show this to be an issue getting complete data. Total MVPA was calculated for both weekday and weekend days as well as during school hours using the Actilife segment feature. Data were downloaded using Actilife Software. Wear time was classified using the Choi et al. [35] algorithm. To adjust for wear time, percent of time spent in sedentary, light physical activity, and MVPA was also reported. A valid day had at least 10 hours of data. To be included in the study participants had to have at least 3 weekdays and one weekend day of valid data.

### 2.4. Statistical Analysis

Descriptive statistics were calculated for time in MVPA on weekdays and the weekend. Means and standard deviations were calculated for PACER, curl-up, push-up, and two-site skinfold. Percentage of youth in the Healthy Fitness Zone (HFZ) for each component was also calculated.

## 3. Results

**Figure 1:**
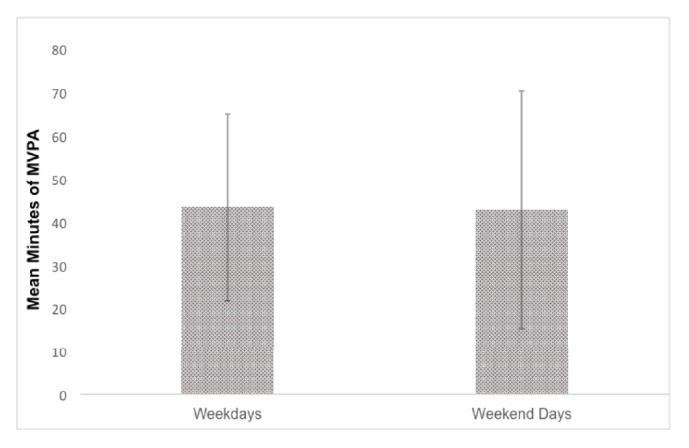
Minutes of MVPA on weekdays and weekend days.

**Figure 2:**
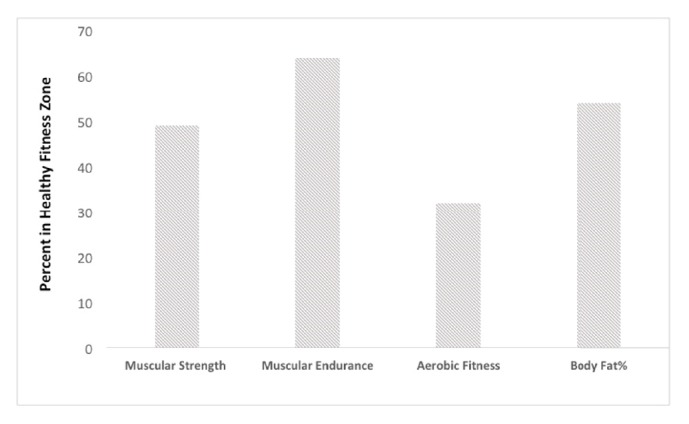
Percent of adolescents in the Healthy Fitness Zone for FITNESSGRAM test battery.* Note.* For muscular strength, Healthy Fitness Zone is ≥ 18 push-up repetitions for males and ≥ 7 push-up repetitions for females; for muscular endurance, Healthy Fitness Zone is ≥ 24 curl-up repetitions for males and ≥ 18 curl-up repetitions for females; for aerobic capacity, Healthy Fitness Zone is ≥ 44.3 mL/kg/min for males and ≥ 38.8 mL/kg/min for females; for % body fat, Healthy Fitness Zone is ≤ 22.3% in males and ≤ 31.3% in females.

Table 1 presents the participant characteristics as well as percent sedentary times, light physical activity, and MVPA for the period before, during, and after school hours.  Figure 1 shows youth MVPA on weekdays and weekends. Adolescents averaged 43.3±21.6 minutes of MVPA per weekday compared to 42.7±27.5 per weekend day. For absolute minutes per day of MVPA, during school hours, adolescents accumulated 17.1±9.0 minutes of MVPA compared to 5.9±3.4 minutes before school hours and 21.0±13.6 minutes after school hours.  Figure 2 shows the percentage of youth in the HFZ for each component of the FITNESSGRAM. Adolescents averaged 18.9±11.0 repetitions for push-ups (49% Healthy Fitness Zone; HFZ), 44.5±26.4 repetitions for curl-ups (64% HFZ), 34.7±24.8 PACER laps (32% HFZ), and 22.1%±10.0% percent body fat (54% HFZ).

## 4. Discussion

The purpose of the study was to explore the physical activity and several components of health-related fitness of adolescents in two secure Juvenile Justice facilities. Adolescents within the Juvenile Justice System are falling short of the recommended 60 minutes of MVPA per day and 30 minutes of MVPA during school hours and are also in need of improving their health-related fitness, especially cardiorespiratory endurance, where only 1 in 3 youth was in the HFZ.

Adolescents in custody accumulate slightly less daily physical activity (MVPA) when compared to adolescents who are not in custody [17, 36–40]. We had anticipated juvenile defenders being more active because of the regimented schedules and available facilities to be physically active. The largest deficit appears to be in the amount during school hours where adolescents in the current study are accumulating just over half the recommended levels. The one area where this population appear to be accumulating more physical activity than other groups is on the weekend where adolescents maintained their physical activity. Previous studies have identified a significant decrease in physical activity on weekends [37, 38]. Even without typical school time physical activity opportunities on weekends in these facilities, adolescents did have opportunities to participate in some recreational activities including basketball, football, and softball, which we hypothesized would contribute to higher weekly physical activity.

Clearly, interventions are needed in this population targeting PA and fitness. Beets and colleagues [41] suggest that physical activity can be increased by expanding, extending, and enhancing opportunities to be active. Correctional/detention facilities should require physical education (PE) as PE often contributes 25% of a student's daily physical activity [37] and 20% of their physical activity accumulated at school [42]. This alone has the potential to help adolescents meet daily recommendations. Keys to physical education implementation in this population might include providing choice and individualizing instruction as well as using curricular approaches such as the health club model or teaching personal and social responsibility [43]. Classroom physical activity interventions have also had some success in increasing both physical activity [44] and behavior [45]. Providing activity breaks or active academics might help increase physical activity but also the behavior and focus of these adolescents in their classrooms. Morning physical activity programs [46] may improve both health and behavior. This could be as simple as a walking club [46]. Adolescents have downtime in the morning around breakfast, so it seems that morning activities could be easily implemented. After school [47] and intramural [48] programming could also be beneficial. On weekends in these facilities, both pick-up and intramural games like softball and basketball were often played which allowed for higher levels of physical activity. Adolescents who did not choose to participate could engage in an unstructured activity of their choice or spend time in their individual units. Multicomponent physical activity programs called CSPAP (Comprehensive School Physical Activity Program) [49] have also shown improvements in physical activity [50], cardiorespiratory endurance [23], and cardiometabolic outcomes [51] as well as classroom behavior [12]. CSPAPs use all available resources during, before, and after school hours for children and adolescents to be active and meet the 60 minutes of physical activity per day guideline. CSPAPs usually involve five components including quality physical education, providing additional physical activity opportunities before, during, and after school hours, and facilitating community, staff, and family engagement. For these types of programs to be effective, additional training is likely required for both school teachers and facility security staff. Although not something we specifically looked at in this study, these types of interventions of Juvenile Justice facilities may also help protect youth from cardiovascular health issues that are often seen in youth with adverse childhood experiences [52].

To our knowledge this is the first study to examine objective measures of physical activity and fitness of adolescents in custody. However, this study is not without limitations. The generalizability of the study is limited due to the facilities being only from one state. The study also had a relatively small sample size, although many secure Juvenile Facilities have small numbers. Furthermore, the physical activity and school structure may be different across other facilities. Future research should also contextualize what activities youth participated in and how often. Additionally, a vast majority of adolescents in secure facilities are male. For example, at the time of this study, only 8 girls statewide were in secure facilities whereas approximately 100-150 males were in secure facilities at given time. Finally, FITNESSGRAM testing was completed the week prior to physical activity measurement to ensure that the PACER test did not influence daily physical activity patterns although the testing could have made students aware of their fitness levels, which may have influenced physical activity.

## 5. Conclusions

Adolescents in custody, like most adolescents, could benefit from additional physical activity and fitness opportunities. These facilities may need to redefine the training and roles of school and facility personnel in order to increase opportunities. Partnering on programming with local organizations or universities [53] may help fulfil the training or expertise needs to start these programs.

## Figures and Tables

**Table 1:** Participant characteristics, school segment sedentary times, and school segment physical activity data for the total sample.

		Mean or Percent	Standard Deviation
Participant	Age (years)	17.1	1.0
Characteristics			

	White	52%	

	Hispanic	33%	

	African American	7%	

	Pacific Islander	4%	

	American Indian	4%	

Before School	Sedentary time	87.3%	6.3%
Behavioral Data			

	Light Physical Activity	9.4%	5.0%

	Moderate-to-Vigorous	3.3%	1.7%
	Physical Activity		

During School	Sedentary time	88.2%	5.4%
Behavioral Data			

	Light Physical Activity	8.8%	5.5%

	Moderate-to-Vigorous	3.0%	2.8%
	Physical Activity		

After School	Sedentary time	87.1%	8.9%
Behavioral Data			

	Light Physical Activity	9.2%	6.2%

	Moderate-to-Vigorous	3.7%	2.9%
	Physical Activity		
